# Sex-related differences in vitamin D testing in the Veneto Region, Italy: a retrospective analysis from 2005 to 2016

**DOI:** 10.1007/s11657-024-01460-w

**Published:** 2024-10-30

**Authors:** Sandro Giannini, Annalisa Pitino, Stefania Sella, Maria Fusaro, Gaetano Paride Arcidiacono, Marco Onofrio Torres, Martina Zaninotto, Mercedes Gori, Andrea Aghi, Colin Gerard Egan, Paolo Simioni, Giovanni Tripepi, Mario Plebani

**Affiliations:** 1 Clinica Medica 1, Department of Medicine, University of Padova, Padua, Italy; 2 Institute of Clinical Physiology (IFC), National Research Council (CNR), Rome, Italy; 3 Institute of Clinical Physiology (IFC), National Research Council (CNR), Pisa, Italy; 4 QI.Lab.Med, Spin-off of the University of Padova, Padua, Italy; 5 CE Medical Writing SRLS, Pisa, Italy; 6 Institute of Clinical Physiology (IFC), National Research Council (CNR), Reggio Calabria, Italy

**Keywords:** Osteoporosis, Vitamin D, Supplementation, Sex

## Abstract

***Summary*:**

A retrospective analysis was performed to evaluate the frequency of vitamin D blood testing in individuals from the Padua province, Veneto, Italy from 2005 to 2016. A significant increase in the frequency of vitamin D blood tests, particularly in females was observed and in individuals with severe vitamin D deficiency (Class I).

**Purpose:**

Vitamin D deficiency has been linked to negative health outcomes that extend beyond bone-related conditions. The frequency of vitamin D blood testing in residents from the Padua province, (Veneto, Italy) from 2005 to 2016 was evaluated.

**Methods:**

Data were retrospectively retrieved from blood test databases (Laboratory Medicine Unit, Padua University Hospital) and information on number of vitamin D blood tests performed on residents from 2005 to 2016 was collected. Data were stratified by sex and ten birth cohorts from 1901 to 2016. Blood tests were classified into five vitamin D classes: I < 50 nmol/L, II 50–74.9 nmol/L, III 75–149 nmol/L, IV 150–250 nmol/L, and V > 250–1000 nmol/L. Blood test trends were analyzed as blood test rate and vitamin D class rate/resident population. Population analysis was analyzed by incidence rates and stratified by vitamin D class.

**Results:**

293,013 vitamin D blood tests were conducted between 2005 and 2016 across 10 birth cohorts. Females accounted for 75% of tests and fewer were conducted in the youngest and oldest birth cohorts. Sex differences in vitamin D blood test frequency were observed; adjusted rates ranging from 1.7 to 35.6% for males and 8 to 81% for females from 2005 to 2016. Crude incidence rates (per 1000 from 2005 to 2016) varied from 1.5 to 10.8‰ for males and 7 to 19.4‰ for females. Crude blood test rates for vitamin D deficiency (Class I) increased from 1.1 to 9.9‰ in 2016 for males and 5 to 17.3‰ for females. Crude incidence rates (from 2005 to 2016) for Class I were 9.7–57.1‰ in males and 43.6–92.4‰ in females.

**Conclusions:**

These findings highlight sex-related differences in vitamin D testing, providing valuable insight for healthcare planning.

**Supplementary Information:**

The online version contains supplementary material available at 10.1007/s11657-024-01460-w.

## Introduction

Vitamin D exists in two bioequivalent forms. Vitamin D2 (D2), known as ergocalciferol, is primarily obtained from sources like fungi, yeast, and oral supplements. Vitamin D3 (D3), known as cholecalciferol, is mainly acquired through skin exposure to ultraviolet B radiation from sunlight, consumption of certain foods such as oily fish, and fortified products (milk, juices, margarines, yogurts, cereals, and soy), as well as oral supplements [1]. Most foods contain extremely low/undetectable levels of vitamin D while wild oily fish contain significantly higher levels (~ 500 IU per 100 g serving) [2]. Once absorbed from the intestine, both D2 and D3 are metabolized in the liver to 25-hydroxyvitamin D [25(OH)D], which comprises 25(OH)D2 and 25(OH)D3. 25(OH)D, also known as calcidiol, is subsequently converted into 1,25-dihydroxyvitamin D [1,25(OH)2D], (calcitriol), primarily in the kidney and some other organs through the action of the 1-hydroxylase enzyme [3]. The main effects of vitamin D are mediated through the endocrine and autocrine actions of calcitriol, which activates the vitamin D receptor in cells, as previously described [4, 5].

Vitamin D is crucial in maintaining musculoskeletal health by regulating calcium and phosphate metabolism [6] and insufficient intake or inadequate levels can disrupt bone metabolism, leading to elevated parathyroid hormone secretion and increased bone resorption [7]. It enhances calcium absorption in the gut, promoting bone mineralization and preventing conditions like rickets in children and osteomalacia in adults [8]. Indeed, the role of vitamin D in enhancing bone health has garnered substantial attention over the past decades [9–11]. Clinical studies aiming to demonstrate the anti-fracture effects of medications for osteoporosis consistently include calcium and vitamin D supplementation [12, 13]. Moreover, research has shown that vitamin D depletion increases the risk of osteoporotic fractures and reduces the efficacy of various commonly used osteoporosis treatments [6, 14]. In clinical practice, the benefits of prescribing vitamin D supplements alongside anti-fracture drugs have been evident, particularly in postmenopausal women. When osteoporosis drugs are combined with vitamin D supplements, these patients experience more significant increases in bone density and a more pronounced reduction in fracture risk compared to those taking osteoporosis drugs alone [15]. Consequently, the Italian Medicines Agency recommends vitamin D supplementation for individuals at risk of fragility fractures or those initiating osteoporosis medication [16].

Vitamin D deficiency is recognized to be associated with adverse health outcomes beyond bone-related issues, including cancer [17, 18], cardiovascular diseases[19], diabetes mellitus [20], autoimmune conditions [21], and neurodegenerative diseases [22], including increased risk of mortality [23]. However, recent meta-analyses and trials have sparked debate on the interpretation of results, affecting our understanding of the role of vitamin D’s in various health conditions [24, 25]

Serum 25(OH)D measurement is commonly used in clinical practice to evaluate vitamin D status [26]. Vitamin D deficiency (specifically, levels of < 50 nmol/L or 20 ng/mL [9, 25]) represents a global concern. Surprisingly, studies investigating vitamin D status across Europe have revealed a higher prevalence of vitamin D deficiency in Southern European countries compared to Northern Europe, despite the greater sunlight exposure in the south [27]. This disparity is particularly evident among the elderly population, such as women with osteoporosis, in sunny countries like Italy and Greece [28, 29].

Recently, studies from the Netherlands [30], UK [31], Australia [32], France [33], and Switzerland [34] reported retrospective analysis on the trends of incidence in the testing for vitamin D. In all of these retrospective analyses, a substantial increase in vitamin D testing was observed [30–34]. To date, no studies have been conducted in Italy.

Since the early 2000s, the Veneto Regional Health System has taken significant steps to address hypovitaminosis D and improve the quality of care for osteoporosis patients. One notable initiative was the establishment of the Regional Center for Osteoporosis, which is affiliated with Padua University Hospitals. This center plays a key role in clinical, epidemiological, and advisory aspects related to osteoporosis. According to this, the current study aimed to analyze changes in the incidence of vitamin D blood tests among residents of Padua from 2005 to 2016 to investigate the specific impact of the various programs implemented in the Veneto Region and to understand how these programs may have influenced the test incidence.

## Methods

### Data collection

Data were retrieved from blood test databases (for an audit on the appropriateness of laboratory test request) of the Laboratory Medicine Unit, University Hospital of Padua, an Italian province, which accounts for approximately 19% (~ 1 million) of the Veneto population.

Data on the number of blood tests on vitamin D undertaken on residents spontaneously referring to the above-mentioned Unit from 2005 until 2016 were considered.

Data were differentiated by sex and ten birth cohorts: #1, 1901–1925; #2, 1926–1935; #3, 1936–1945; #4, 1946–1955; #5, 1956–1965; #6, 1966–1975; #7, 1976–1985; #8, 1986–1995; #9, 1996–2005; and #10, 2006–2016. Blood test results were classified into five classes, according to 25(OH)D vitamin D blood levels: Class I < 50 nmol/L; II 50–74.9 nmol/L; III 75–149 nmol/L; IV 150–250 nmol/L; and V > 250–1000 nmol/L.

This study was approved on 14/03/2019 by the Ethics Committee of the Regione del Veneto-Azienda Ospedaliera di Padova, Per La Sperimentazione Clinica Della Provincia di Padova, Italy (Protocol no. 0030936). This study was performed in accordance with the ethical standards laid down in the 1975 Declaration of Helsinki.

### Vitamin D assay

Serum 25(OH)D was measured using the automated immunochemiluminescent method, with a LIAISON® 25 OH Vitamin D TOTAL Assay 310,600 (DiaSorin Inc., Stillwater, MN, USA). Sensitivity was < 10 nmol/L, and the intra-assay coefficients of variation (CV) were between 2.9 and 5.5%, while inter-assay CV was 6.3–12.9%. This method has been validated, and the quality and comparability of results over time has been regularly monitored through internal quality control and external quality assurance programs, being the laboratory accredited according to the ISO15189 international standard.

### Statistical analysis

Two separate analyses were performed: the first evaluating the temporal trend in blood tests and the second on the population trend.

Blood test trends were analyzed based on the rate of blood tests and the distribution of vitamin D levels among residents. To evaluate these trends, the analysis considered both crude and adjusted rates (using direct standardization whereby a reference population was used as the standard population) by birth cohort on residents for the year 2006 [36]. Population trends were assessed by calculating overall incidence rates and incidence rates stratified by vitamin D levels. All analysis was stratified by sex.

Trend analysis was performed by jointpoint regression [37] (Jointpoint Regression Program, version 4.6.0 provided by the Surveillance, Epidemiology, and End Results Program of National Cancer Institute [38].

Jointpoint regression was performed on the prevalence of blood analysis trends, and on the incidence of people who submitted a blood test. This methodology is usually applied to identify if there are significant points (joints) of change in trends. The slope of each continuous linear phase was viewed as the period percent change in prevalence.

Due to sex differences in analysis prescription reported in the literature, the analysis was further stratified by sex.

Reported prevalence (calculated by dividing the number of individuals submitting a vitamin D blood tests during the follow-up period by the number of patients in the study population and expressed as a percentage) was weighted using the Paduan resident population in 2006 for the overall trend by sex and standardized by birth cohorts. Reported incidence was calculated as the number of new blood tests from 1000 patients per year. After estimating analysis prescription prevalence in each cohort-sex group (for each period), or prevalent/incident people, the permutation test for jointpoint regression was used to detect significant annual percent change (APC). Models were fitted using the log scale and assumed heteroscedasticity of observations using the standard errors as weights and assumed uncorrelated errors.

A *p*-value of < 0.05 was considered statistically significant. Given the relatively long time period (12 data points), a default number of jointpoints of min 0-max 2 was chosen. Apart from jointpoint regression, all other analysis was carried out by STATA 16, StataCorp, Lakeway Drive, College Station, TX, USA.

## Results

### Study population

This retrospective analysis encompassed an extensive dataset of 293,013 vitamin D blood tests conducted over the period from 2005 to 2016, representing individuals from 10 distinct birth cohorts. It was observed that a significant proportion of tests (approximately 75% of the total number), were administered to females (Table 1).

**Table 1:** Vitamin D blood tests conducted over the period from 2005 to 2016 stratified by gender and ten birth cohorts

	Birth cohort	2005	2006	2007	2008	2009	2010	2011	2012	2013	2014	2015	2016	Total
Male	1	120 (16)	184 (17)	206 (14)	224 (10)	315 (10)	385 (8)	479 (7)	398 (5)	347 (4)	290 (3)	223 (2)	166 (1)	3337 (4)
	2	210 (28)	296 (27)	361 (25)	453 (21)	658 (21)	972 (20)	1191 (19)	1338 (17)	1333 (14)	1577 (14)	1509 (12)	1370 (10)	11,268 (15)
	3	158 (21)	219 (20)	339 (24)	370 (17)	562 (18)	1027 (21)	1342 (21)	1616 (20)	1952 (21)	2351 (21)	2755 (22)	2528 (19)	15,219 (20)
	4	118 (15)	166 (15)	201 (14)	293 (14)	511 (16)	719 (15)	976 (15)	1262 (16)	1575 (17)	2054 (18)	2459 (19)	2759 (21)	13,093 (18)
	5	50 (7)	105 (10)	154 (11)	382 (18)	561 (18)	719 (15)	1011 (16)	1332 (17)	1484 (16)	1830 (16)	2162 (17)	2351 (18)	12,141 (16)
	6	32 (4)	48 (4)	66 (5)	201 (9)	334 (10)	485 (10)	613 (10)	844 (11)	951 (10)	1222 (11)	1424 (11)	1624 (12)	7844 (11)
	7	20 (3)	31 (3)	35 (2)	83 (4)	122 (4)	197 (4)	280 (4)	367 (5)	464 (5)	649 (6)	734 (6)	876 (7)	3858 (5)
	8	20 (3)	29 (3)	28 (2)	72 (3)	58 (2)	131 (3)	169 (3)	228 (3)	283 (3)	366 (3)	426 (3)	512 (4)	2322 (3)
	9	34 (4)	25 (2)	33 (2)	35 (2)	54 (2)	98 (2)	216 (3)	277 (3)	478 (5)	559 (5)	595 (5)	581 (4)	2985 (4)
	10	0 (0)	0 (0)	11 (1)	29 (1)	30 (1)	50 (1)	146 (2)	261 (3)	386 (4)	489 (4)	517 (4)	528 (4)	2447 (3)
	*Total*	*762 (100)*	*1103 (100)*	*1434 (100)*	*2142 (100)*	*3205 (100)*	*4783 (100)*	*6423 (100)*	*7923 (100)*	*9253 (100)*	*11,387 (100)*	*12,804 (100)*	*13,295 (100)*	*74,514 (25)*
Female	1	546 (15)	687 (13)	749 (12)	859 (11)	1431 (12)	1627 (10)	1649 (8)	1530 (7)	1370 (5)	1174 (4)	915 (3)	725 (2)	13,262 (6)
	2	1064 (29)	1467 (28)	1704 (28)	2005 (26)	2925 (24)	3616 (22)	4068 (20)	4317 (18)	4411 (17)	4507 (15)	4268 (13)	3891 (12)	38,243 (18)
	3	1004 (27)	1433 (27)	1729 (28)	2081 (27)	3108 (25)	4175 (25)	5104 (25)	5687 (24)	6118 (23)	6700 (22)	6782 (21)	6621 (20)	50,542 (23)
	4	669 (18)	1094 (21)	1202 (20)	1546 (20)	2551 (21)	3301 (20)	4242 (21)	4789 (20)	5335 (20)	6279 (21)	6561 (20)	6708 (20)	44,277 (20)
	5	249 (7)	364 (7)	442 (7)	768 (10)	1297 (11)	2177 (13)	3081 (15)	3790 (16)	4635 (17)	5778 (19)	6415 (20)	6782 (20)	35,778 (16)
	6	79 (2)	99 (2)	163 (3)	283 (4)	495 (4)	884 (5)	1297 (6)	1740 (7)	2362 (9)	3144 (10)	3763 (12)	4357 (13)	18,666 (9)
	7	26 (1)	42 (1)	65 (1)	109 (1)	240 (2)	365 (2)	572 (3)	718 (3)	913 (3)	1247 (4)	1596 (5)	1866 (6)	7759 (4)
	8	17 (0)	22 (0)	32 (1)	40 (1)	99 (1)	199 (1)	278 (1)	382 (2)	528 (2)	726 (2)	867 (3)	988 (3)	4178 (2)
	9	32 (1)	44 (1)	47 (1)	49 (1)	58 (0)	93 (1)	174 (1)	323 (1)	481 (2)	642 (2)	738 (2)	800 (2)	3481 (2)
	10	0 (0)	7 (0)	13 (0)	20 (0)	29 (0)	54 (0)	130 (1)	246 (1)	374 (1)	421 (1)	498 (2)	521 (2)	2313 (1)
	*Total*	*3686 (100)*	*5259 (100)*	*6146 (100)*	*7760 (100)*	*12,233 (100)*	*16,491 (100)*	*20,595 (100)*	*23,522 (100)*	*26,527 (100)*	*30,618 (100)*	*32,403 (100)*	*33,259 (100)*	*218,499 (25)*
													*Total*	*293,013(100)*

Data are presented as number and percentage (%). Birth Cohort: 1, 1901–1925; 2, 1926–1935; 3, 1936–1945; 4, 1946–1955; 5, 1956–1965; 6, 1966–1975; 7, 1976–1985; 8, 1986–1995; 9, 1996–2005; 10, 2006–2016

It is worth highlighting that within the birth cohort, the #3 (1936–1945) cohort emerged as that cohort where the highest number of tests was undertaken. Specifically, cohort #3 accounted for 20% of the total number of tests among females and 23% among males.

The second most frequently tested cohorts were #4, #5, and #2, demonstrating similar frequencies for both males (18%, 16%, and 15%, respectively) and females (20%, 16%, and 18%, respectively). Interestingly, there was a trend of fewer tests administered to the youngest birth cohorts (born after 1966) as well as the oldest cohort (1901–1925) (Table 1).

### Sex-related differences in blood test rates

**Fig. 1:**
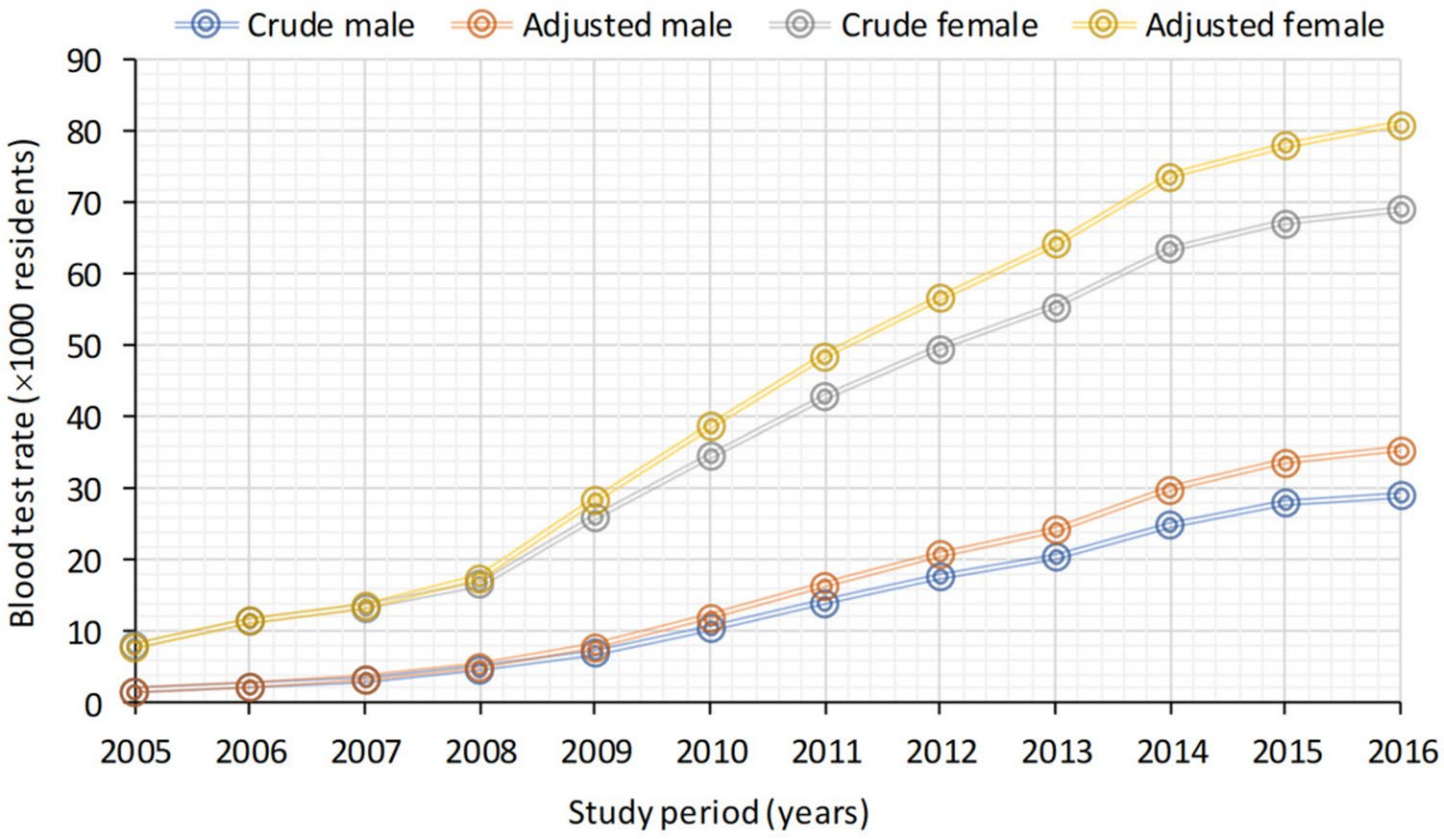
Crude and adjusted blood test prevalence rates (× 1000) on male and female residents for the years 2005–2016. Prevalence of blood test rates was calculated as the number of blood tests expressed as a percentage

Crude and adjusted rates of blood tests administered to male and female individuals for each year are presented in Fig. 1. A notable shift in the analysis emerged, whereby a marked increase in adjusted rates from 2009 onwards among males and from 2008 among females was observed in comparison to crude rates.

Overall, a distinct sex-related disparity was apparent in the frequency of vitamin D blood tests, with a higher prevalence observed in females compared to males. This sex-specific gap widened further over subsequent years. When considering adjusted rates, the disparity ranged from 1.7% (95% CI 1.6–1.8) to 35.6% (95% CI 35–36.1) for males and from 8% (95% CI 7.7–8.3) to 81% (95% CI 80.2–81.8) for females, spanning the years from 2005 to 2016 (Table S1).

Jointpoint regression analysis was used to assess the goodness of fit for the observed blood test rates adjusted by birth cohort, as well as the estimated rates. This analysis confirmed a significant annual increase in blood test rates for both sexes, as depicted in Fig. 1S from 2005 to 2016. Among males, jointpoint analysis revealed two clear transition points, the first occurring in 2011 and the second in 2014 (Fig. S1A).

These transitions corresponded to distinct APCs in blood test rates: an initial increase of 47.2% from 2005 to 2011, followed by 21.2% from 2011 to 2014, and a subsequent 9.3% increase from 2014 onwards. In contrast, among females, a single jointpoint in 2011 divided the trend into two distinct periods. From 2005 to 2011, blood test rates exhibited a substantial annual increase of 37.3%, while from 2011 onwards, the annual increase was 10.5% (Fig. S1B).

### Sex-related differences in incidence rates of blood tests for vitamin D

**Fig. 2:**
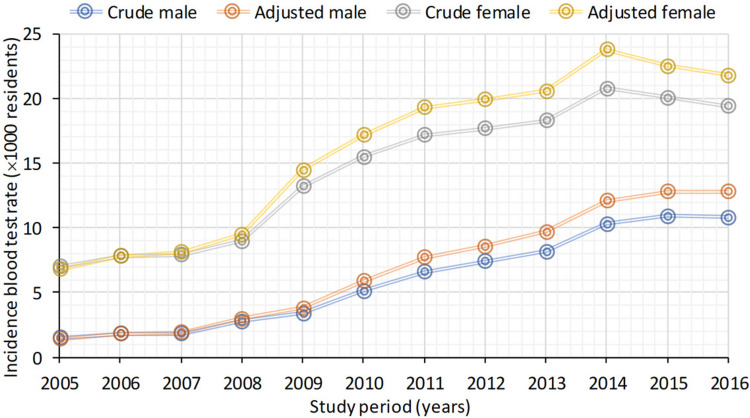
Crude and adjusted incidence of blood test rates (× 1000) on male and female residents for the years 2005–2016. Incidence was calculated as the number of new blood tests from 1000 patients per year

When considering the incidence rates of people who submitted blood tests for vitamin D, crude rates increased from 1.5‰ (95% CI 1.4–1.6) to 10.8‰ (95% CI 10.5–11.1) for males and from 7‰ (95% CI 6.8–7.3) to 19.4‰ (95% CI 19.0–19.8) for females, with significant difference between crude and adjusted rates (Fig. 2, Table S2).

The year of 2011 emerged as the jointpoint for both sexes. For males in the previous period, there was an annual significant increase of 36.4% and of 12.1% thereafter. For females, there was an annual increase of 21.4% until the year 2011 (Fig. S2). For males, all trends, blood rates, and incidence values were observed to increase over time compared to females.

### Blood test rates and trends across different vitamin D classes

**Fig. 3:**
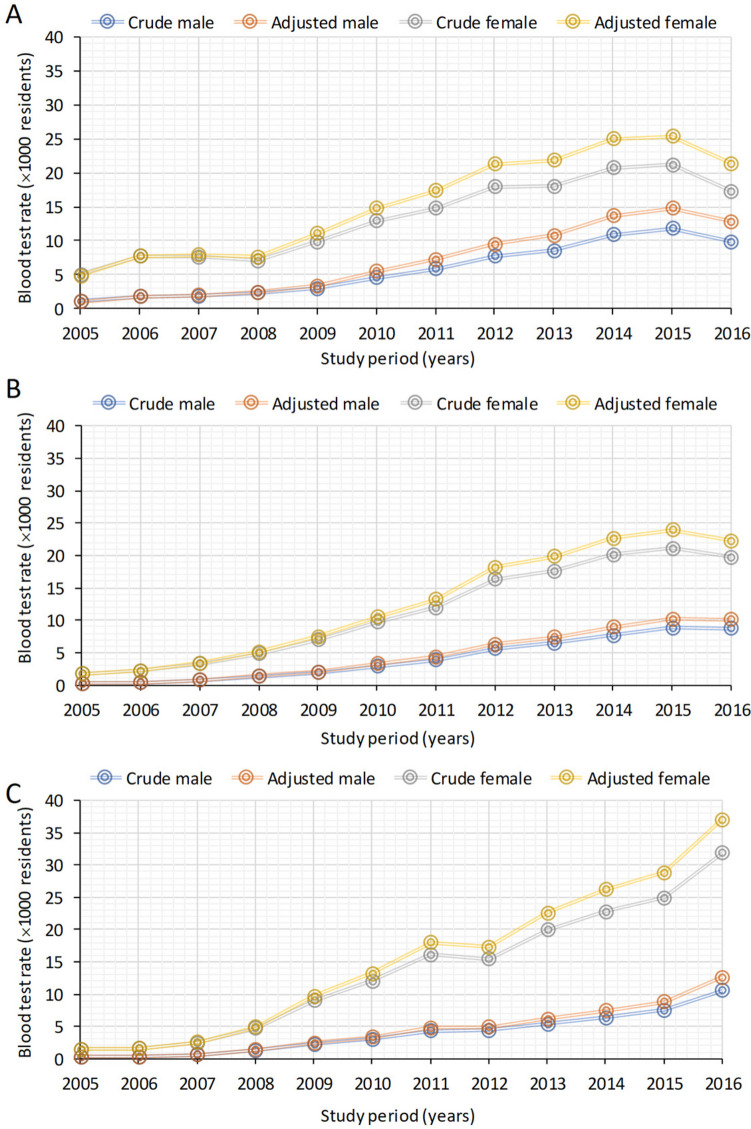
Crude and adjusted blood test prevalence rates (× 1000) on male and female residents for the years 2005–2016 by vitamin D classes (I, II, III). Class I =  < 50 nmol/L **A**, Class II = 50–74.9 nmol/L **B**, and Class III = 75–149 nmol/L **C**

Crude blood test rates for vitamin D deficiency (Class I) increased from 1.1‰ in 2005 to 9.9‰ in 2016 for males and from 5 to 17.3‰ for females (Fig. 3, Table S3). Classes II and III were also observed to increase over time, with crude rates increasing from 0.3 to 8.7‰ and from 0.3 to 10.5‰ for males, while for females, crude rates escalated from 1.7 to 19.7‰ and from 1.4 to 31.9‰.

The rates of Class I were consistently higher than those of Class II and above until 2015 for males and 2013 for females. Jointpoint regression (Fig. S3) revealed an overall increase in blood test rates across various vitamin D classes. Notably, for both sexes, the rates of Classes II and III exhibited a more substantial increase than that of Class I.

Different jointpoints were evident for the various vitamin D classes. Specifically, for analysis results in Class I, 2014 marked the year of jointpoint, while for Class II, it was 2012, and for Class III, it was 2009 and 2010, respectively, for males and females. Data for Class IV and V was not considered due to low numbers.

### Incidence rates in blood tests across different vitamin D classes

**Fig. 4:**
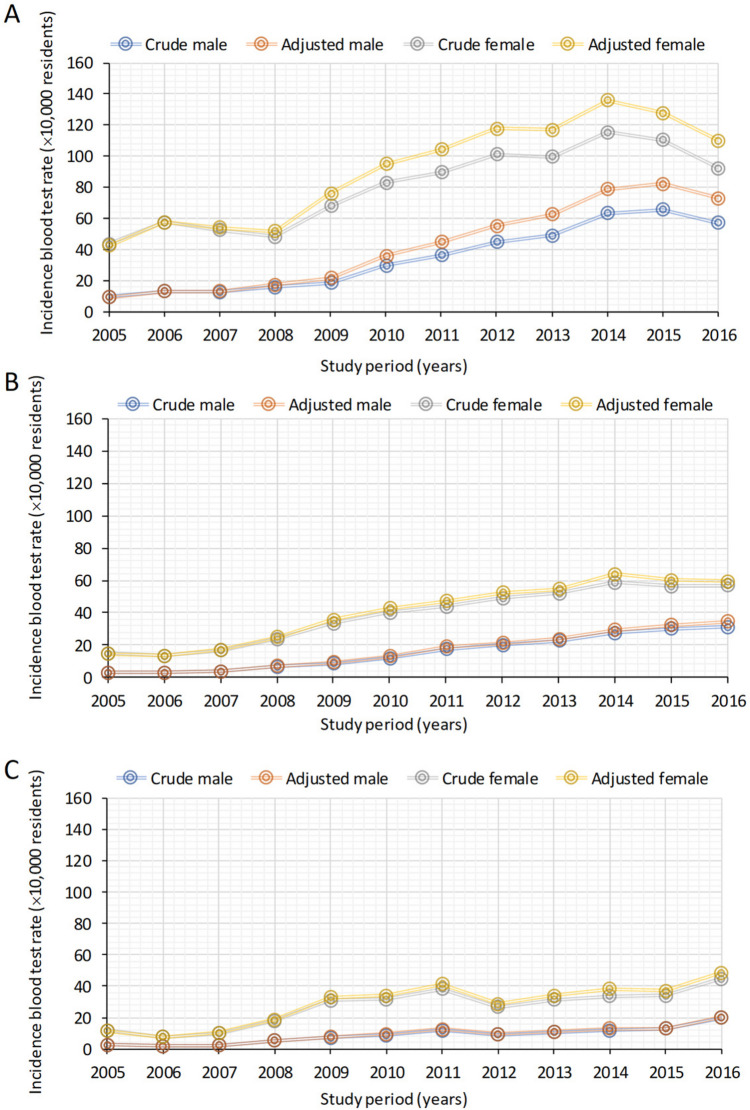
Crude and adjusted incidence rates of (× 10,000) male and female residents for the years 2005–2016 by vitamin D classes (I, II, III) for the years 2005–2016. Class I =  < 50 nmol/L **A**, Class II = 50–74.9 nmol/L **B**, and Class III = 75–149 nmol/L **C**

When considering the incidence of individuals undergoing blood tests, the range of cases indicating vitamin D deficiency (Class I) saw a notable increase, with crude rates ranging from 9.7 to 57.1‰ (× 10,000) in males and between 43.6 and 92.4‰ (× 10,000) in females. Importantly, these rates consistently exceeded those of other vitamin D classes (Fig. 4, Table S4). In this case as well, data for Classes IV and V was not considered due to low numbers. Jointpoint regression analysis confirmed a substantial increase in the incidence of vitamin D deficiency (Class I) in both males and females, but only until the year 2014 (Fig. S4). However, this analysis revealed a notable increase in the number of individuals undergoing blood tests across all vitamin D categories, with the exception of Class V (data not shown).

## Discussion

This study examined 293,013 vitamin D blood tests conducted from 2005 to 2016, involving 10 birth cohorts. Overall, vitamin D testing was observed to substantially increase from 2005 to 2016. This increase may be due, in part, to an increased awareness of the problem of vitamin D deficiency combined with the important benefits of vitamin D for osteoporosis as well as in other settings [6, 39, 40]. Although a generalized increase in vitamin D testing was observed, this increase was not similar among males and females, with significant sex disparity observed; more tests were conducted on females and this difference was observed to increase over time. Incidence rates for vitamin D blood tests increased steadily, but only until 2011 for females. Blood test rates for vitamin D deficiency (Class I) significantly increased for both males and females from 2005 to 2016. Class II and III also showed substantial growth. Incidence rates for vitamin D deficiency (Class I) notably increased for both sexes, consistently surpassing other vitamin D classes.

To the best of our knowledge, this is the first epidemiological study conducted on an Italian cohort, evaluating the incidence of vitamin D testing across different age populations over a 12-year period. Other analogous investigations have been performed in the UK from 2005 to 2015 [31], in Switzerland from 2012 to 2018 [34], in France from 2008 to 2013 [33], and in the Netherlands from 2015 to 2019 [30]. Our findings are in line with these prior studies and underscore a higher prevalence of vitamin D testing among females compared to males.

In 2010, an estimated 22 million women aged over 50 in Europe received a diagnosis of osteoporosis [41]. In Italy, in particular, osteoporosis affects about 5 million subjects, with 80% comprising post-menopausal women [42]. The prevalence of osteoporosis continues to rise, along with the number of associated fragility fractures. Notably, between 2010 and 2020, there was an estimated 23% increase in osteoporosis diagnoses in Europe and a 25% increase in Italy [41].

In a modelling study by Piscitelli et al. undertaken 10 years ago [43], the epidemiological burden of postmenopausal osteoporosis in Italy from 2010 to 2020 was estimated based on national data from the Italian National Institute for Statistics (ISTAT) [44]. Using a Markov model, it was predicted that the number of females would increase from 2010 to 2020 by 14.3% and the rate of osteoporotic fractures would increase for this period by 17.8%. These estimates, although restricted to the female population, parallel with a similar increase in vitamin D blood tests undertaken by females from 2011 to 2016 of 10.5% in the present cohort.

While our findings may be tentatively extended to other regions in Italy, a previous analysis based on data from administrative databases of five Italian Local Health Units Furthermore has revealed that as many as 40% of patients with osteoporosis ≥ 50 years with recent fragility fracture were not receiving osteoporosis medication [13]. These same authors also evaluated the economic burden of osteoporotic patients with fractures and they demonstrated that healthcare costs for osteoporotic patients with fractures were lower in the treated group compared to those left untreated, reinforcing the importance of early diagnosis and appropriate treatment [45]. Furthermore, patients receiving supplementation with calcium/vitamin D saw even lower costs, demonstrating the benefits of these interventions. This insight contributes to the understanding of why vitamin D tests are more frequently administered to women, given the link between vitamin D, osteoporosis, and the importance of prevention and management.

The incidence of vitamin D testing has risen significantly over the years also in the male population. This trend can be attributed to the growing recognition of vitamin D’s pivotal role, not only in bone health but also in a wide spectrum of physiological processes, which can profoundly impact overall health outcomes [25, 39]. Lower levels of vitamin D have been linked to cardiometabolic diseases [46], diabetes [47], and obesity [48]. Furthermore, vitamin deficiency is associated with increased cardiovascular risk [49], especially among females, as it affects the renin–angiotensin–aldosterone system, potentially leading to vascular dysfunction and hypertension.

The potential of vitamin D extends to tumor suppression, anti-inflammation, and immune regulation [39, 50]. Recently, studies have even reported a protective effect of vitamin D supplementation against severe forms of COVID-19, particularly in older adults [50, 51]. The increasing incidence of cardiometabolic and cardiovascular risk factors, particularly among elderly individuals, has amplified the importance of monitoring vitamin D levels. These factors, along with its implications for osteoporosis, have prompted various healthcare systems to advocate for vitamin D supplementation, particularly in elderly individuals[52], as seen in regions such as Veneto. Collectively, these reasons may explain the significant rise in the incidence of vitamin D testing, not only among women but also among men, as highlighted in our study and similar studies conducted in other countries [30–34].

Despite significant advances and increased awareness, vitamin D testing was observed to be markedly lower compared to females in our cohort. There are several reasons that may explain these differing trends. It is recognized that circulating levels of vitamin D are generally higher in males compared to females across age and BMI class as well as individuals with and without osteoporosis [53]. In Italy, these baseline differences may have negatively influenced vitamin D testing rates by males. Indeed, males are generally less frequently considered for a diagnosis of osteoporosis compared to females [54]. Despite these sex-related differences, the important fact remains that there is an evident progression in vitamin D testing in males that should be considered a “signal” that cannot be ignored.

To investigate the specific impact of the various programs implemented in the Veneto Region on the incidence of vitamin testing and eventually vitamin D supplementation according to real needs, we stratified blood tests into 5 classes, with Class I comprising individuals with vitamin D levels below 50 nmol/L, the common cutoff for definition of vitamin D deficiency [25], recently adopted by European government agencies and is in line with the European Calcified Tissue Society, which favors a lower 25(OH)D threshold of > 50 nmol/L (> 20 ng/mL) for sufficiency [24]. In the Nonlinear Mendelian Randomization Study [55], the relationship between 25(OH)D and mortality was investigated in a large, prospective cohort of 307,601 individuals (aged 37–73 years) based in the UK. The main finding that emerged was the risk of mortality increased with decreasing 25(OH)D levels below 50 nmol/L (< 20 ng/mL), highlighting values of 25(OH)D below 50 nmol/L (< 20 ng/mL) as a potential area for intervention and values of 25(OH)D above 50 nmol/L (> 20 ng/mL) as a futile area for intervention.

In a meta-analysis including 15 prospective cohort studies comprising a total of 51,239 participants and 3386 hip fractures [56], individuals with a 25(OH)D concentration lower than 60 nmol/L were observed to have an increased risk of hip fracture, and this cutoff is close to the critical threshold (50 nmol/L; 20 ng/mL) identified in the Nonlinear Mendelian Randomization Study [55], further emphasizing the notion that low 25(OH)D concentrations, mortality, and fractures at least in part share a common pathogenetic pathway, as also suggested by the strong link between mortality and fractures in the population [24].

In our study, we found an increase in the number and incidence of vitamin D testing, especially in individuals belonging to Class I, compared to all other classes. This data demonstrates the effectiveness of the public health intervention programs carried out by the Veneto Region because testing has increased in the class where it was most necessary to do so. Studies from the UK, the US, Canada, and Australia suggest that up to 75% of vitamin D testing may be unnecessary [57] and recent guidelines from the Endocrine Society recommend against screening for 25(OH)D [25]. Indeed, the majority of individuals tested for vitamin D in the present study had normal vitamin D levels.

Reducing unnecessary vitamin D testing not only eases the workload for healthcare providers in terms of result follow-ups but also has no adverse effects on patients’ experiences or health outcomes. Additionally, it contributes to cost savings in healthcare expenditures. Interestingly, our data revealed a significant increase in the incidence of vitamin D testing in females, but only until 2011. This could indicate that we may have reached a point of stability or plateau in testing incidence. For example, Bilinsky and Boyages [32], over an 11-year period, described the continuous growth in 25(OH)D testing as “unsustainable.” In this context, our findings align with the principles of a well-implemented public health program.

### Study limitations

Several limitations of this investigation should be acknowledged. First, it lacked comprehensive clinical data, including patients’ medical histories, comorbidities, and lifestyle factors, which could have offered a deeper understanding of the driving factors behind trends in vitamin D testing.

Second, it did not explore potential confounding variables, such as changes in clinical guidelines or public health initiatives related to vitamin D supplementation, which might have influenced testing trends.

Third, medical databases are susceptible to coding errors, data omissions, and inconsistencies that could potentially impact the accuracy of the results. Fourth, the present analysis did not account for potential multiple measurements in a given individual. It is possible that in some individuals with low 25(OH)D levels could have been treated and sometimes re-measured. Last, this study was primarily focused on describing trends and frequencies of vitamin D testing, without exploring the clinical significance or outcomes of these tests. Further analysis to understand the broader implications of these testing patterns is warranted.

## Conclusion

In this extensive retrospective analysis spanning from 2005 to 2016, we observed a significant increase in the frequency of vitamin D blood tests, with a notable sex disparity favoring females. The analysis revealed distinct shifts and trends in blood test rates, particularly in the year 2011. There was a substantial annual increase in the incidence of blood tests, particularly for individuals with severe vitamin D deficiency (Class I).

Our findings highlight the need for a better understanding of the factors driving this trend and the potential implications for public health initiatives.

## Supplementary Information

Below is the link to the electronic supplementary material.

Supplementary file1 (PPTX 580 KB)

Supplementary file2 (DOCX 17 KB)

Supplementary file3 (DOCX 16 KB)

Supplementary file4 (DOCX 19 KB)

Supplementary file5 (DOCX 23 KB)

## Data Availability

Original datasets can be made available from the corresponding author upon specific request.
