# Supplementary material for: Sex-related differences in vitamin D testing in the Veneto Region, Italy: a retrospective analysis from 2005 to 2016
Source: Arch Osteoporos. 2024 Oct 30;19(1):105. doi: 10.1007/s11657-024-01460-w (PMC11525240; doi:10.1007/s11657-024-01460-w)
Supplement: Supplementary file 1 — Supplementary file1 (PPTX 580 KB) [file 11657_2024_1460_MOESM1_ESM.pptx]

## Slide 1
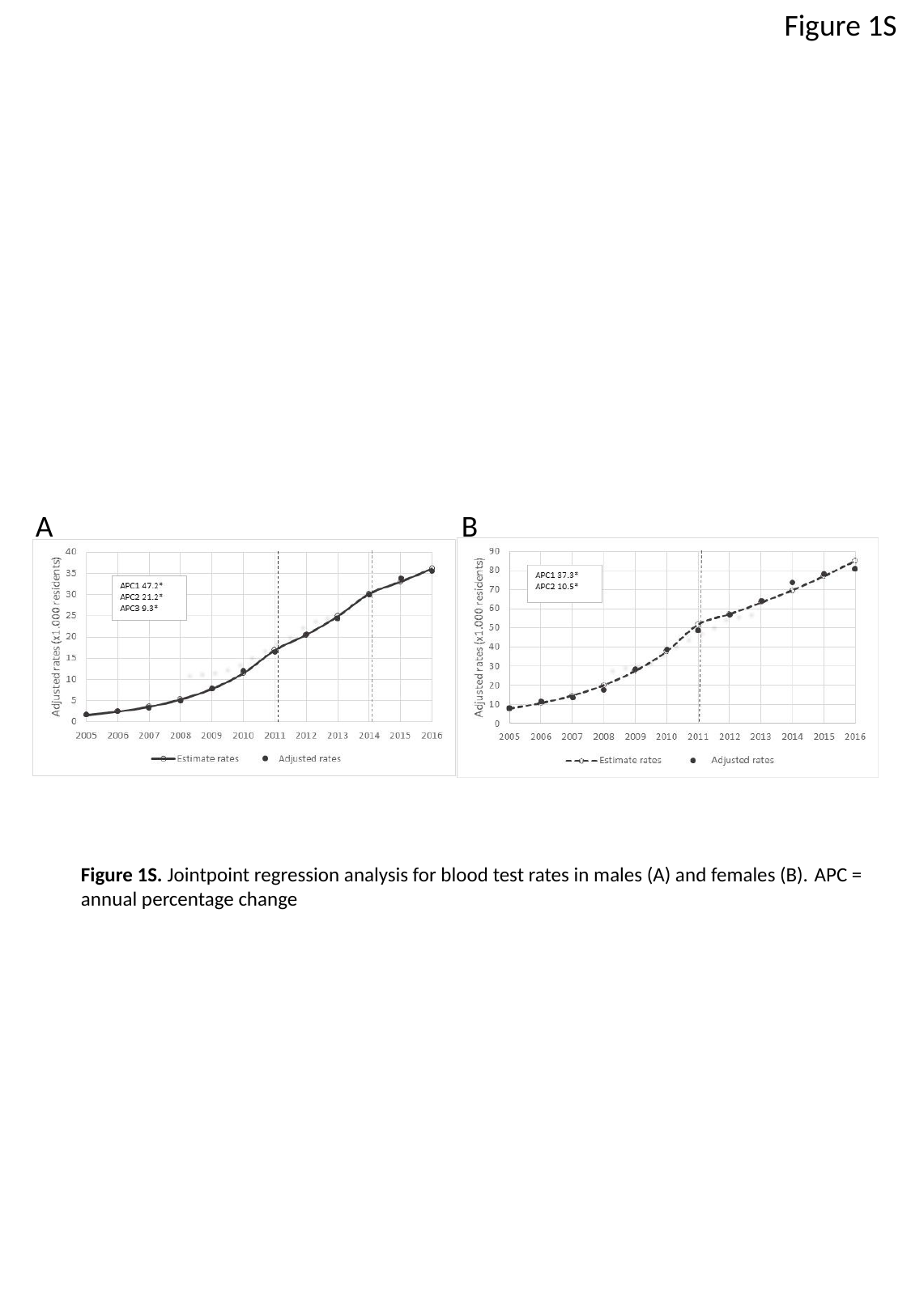

Figure 1S
A
B
Figure 1S. Jointpoint regression analysis for blood test rates in males (A) and females (B). APC =
annual percentage change

## Slide 2
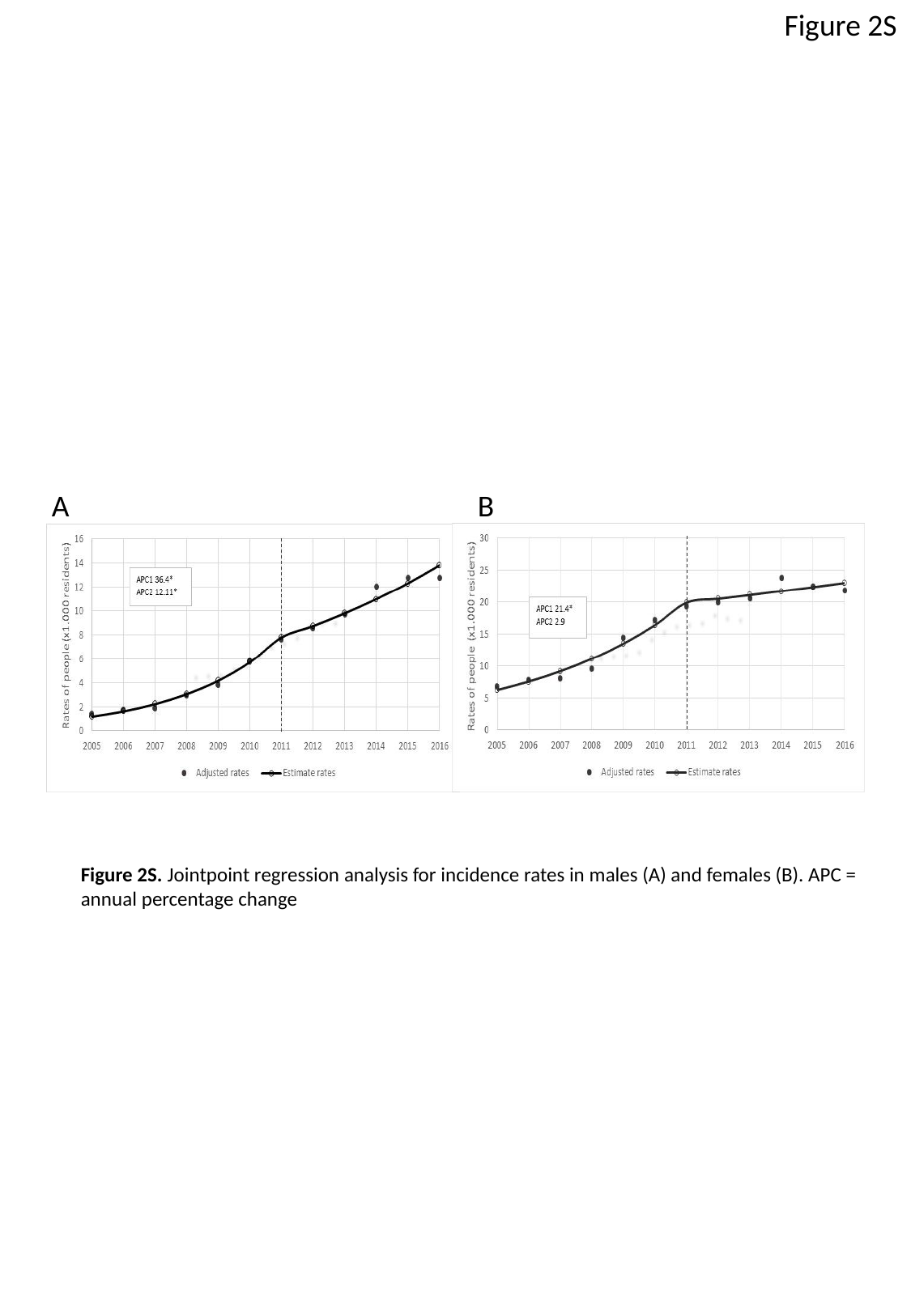

Figure 2S
A
B
Figure 2S. Jointpoint regression analysis for incidence rates in males (A) and females (B). APC =
annual percentage change

## Slide 3
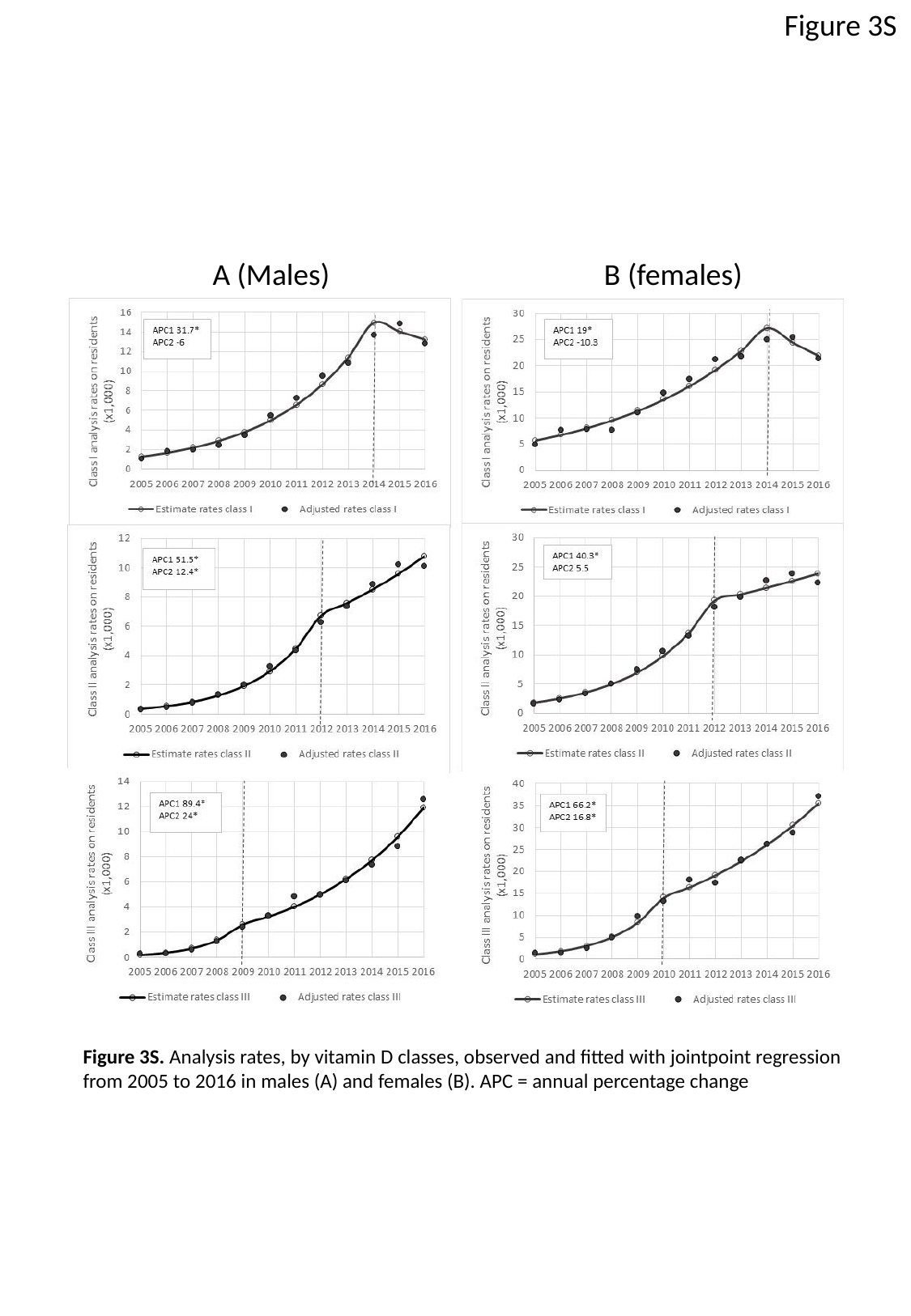

Figure 3S
A (Males) B (females)
Figure 3S. Analysis rates, by vitamin D classes, observed and fitted with jointpoint regression
from 2005 to 2016 in males (A) and females (B). APC = annual percentage change

## Slide 4
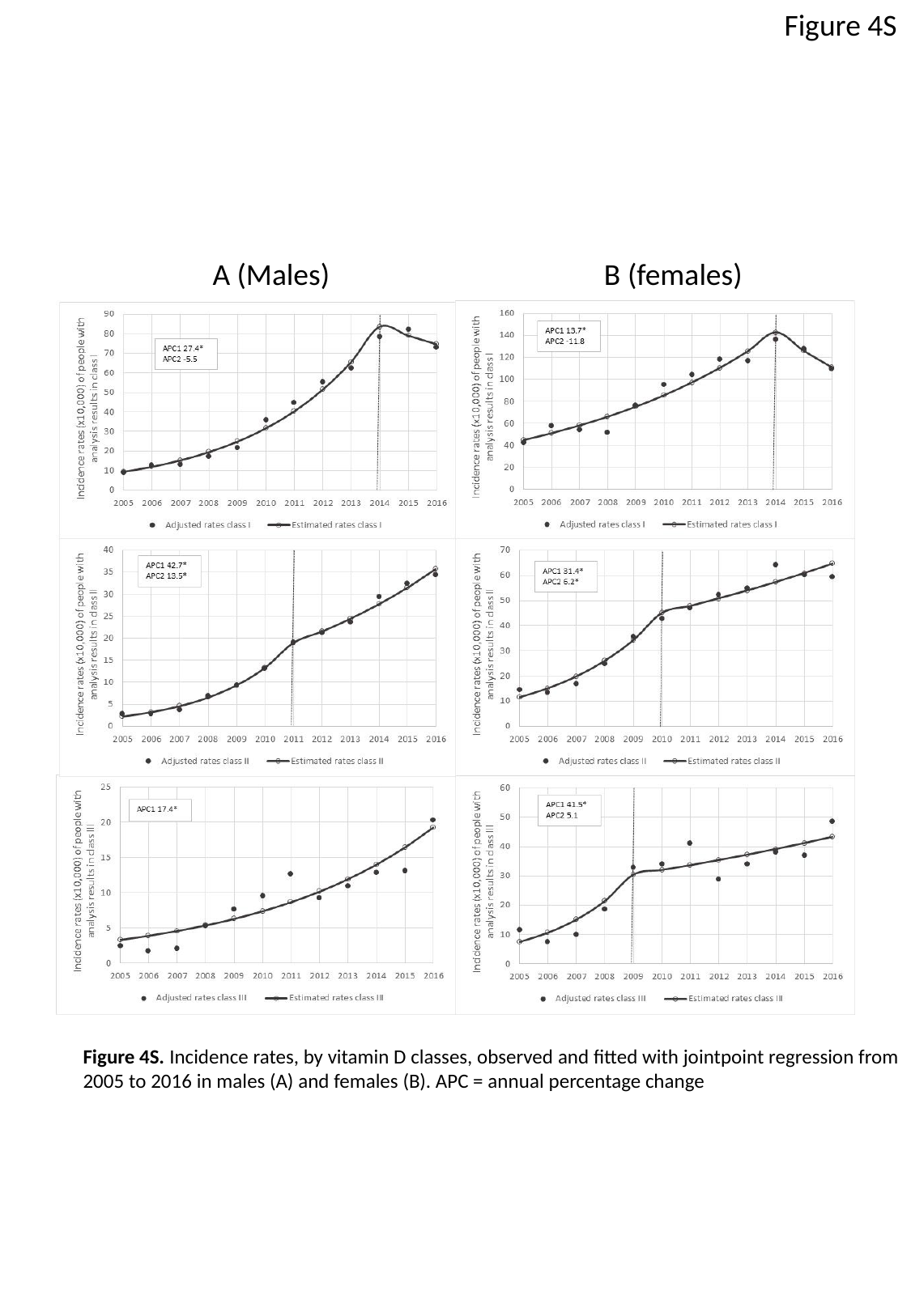

Figure 4S
A (Males) B (females)
Figure 4S. Incidence rates, by vitamin D classes, observed and fitted with jointpoint regression from
2005 to 2016 in males (A) and females (B). APC = annual percentage change
