# Supplementary material for: Sex-related differences in vitamin D testing in the Veneto Region, Italy: a retrospective analysis from 2005 to 2016
Source: Arch Osteoporos. 2024 Oct 30;19(1):105. doi: 10.1007/s11657-024-01460-w (PMC11525240; doi:10.1007/s11657-024-01460-w)
Supplement: Supplementary file 2 — Supplementary file2 (DOCX 17 KB) [file 11657_2024_1460_MOESM2_ESM.docx]

**Table 1S**. Crude and adjusted annual rates for vitamin D blood tests (×1000 residents) stratified by sex.

|  | **Year** | **Population** | **Blood tests** | **Crude rates (CI 95%)** | **Adjusted rates (CI 95%)** |
| --- | --- | --- | --- | --- | --- |
|  |  |  |  |  |  |
| *Males* | 2005 | 430,854 | 762 | 1.8 (1.6-1.9) | 1.7 (1.6-1.8) |
|  | 2006 | 435,091 | 1,103 | 2.5 (2.4-2.7) | 2.5 (2.4-2.7) |
|  | 2007 | 438,812 | 1,434 | 3.3 (3.1-3.4) | 3.4 (3.2-3.6) |
|  | 2008 | 444,609 | 2,142 | 4.8 (4.6-5) | 5.1 (4.9-5.3) |
|  | 2009 | 449,702 | 3,205 | 7.1 (6.9-7.4) | 7.9 (7.6-8.2) |
|  | 2010 | 452,746 | 4,783 | 10.6 (10.3-10.9) | 12.1 (11.8-12.4) |
|  | 2011 | 455,262 | 6,423 | 14.1 (13.8-14.5) | 16.6 (16.2-16.9) |
|  | 2012 | 447,127 | 7,923 | 17.7 (17.3-18.1) | 20.8 (20.4-21.2) |
|  | 2013 | 450,646 | 9,253 | 20.5 (20.1-20.9) | 24.3 (23.9-24.8) |
|  | 2014 | 455,081 | 11,387 | 25 (24.6-25.5) | 30 (29.5-30.5) |
|  | 2015 | 456,229 | 12,804 | 28.1 (27.6-28.5) | 33.9 (33.3-34.4) |
|  | 2016 | 455,528 | 13,295 | 29.2 (28.7-29.7) | 35.6 (35-36.1) |
|  |  |  |  |  |  |
| *Females* | 2005 | 451,925 | 3,686 | 8.2 (7.9-8.4) | 8 (7.7-8.3) |
|  | 2006 | 455,714 | 5,259 | 11.5 (11.2-11.9) | 11.5 (11.2-11.9) |
|  | 2007 | 459,187 | 6,146 | 13.4 (13.1-13.7) | 13.7 (13.4-14.1) |
|  | 2008 | 465,166 | 7,760 | 16.7 (16.3-17.1) | 17.6 (17.2-18) |
|  | 2009 | 471,201 | 12,233 | 26 (25.5-26.4) | 28.4 (27.9-28.9) |
|  | 2010 | 474,984 | 16,491 | 34.7 (34.2-35.2) | 38.8 (38.2-39.3) |
|  | 2011 | 478,954 | 20,595 | 43 (42.4-43.6) | 48.7 (48.1-49.4) |
|  | 2012 | 473,768 | 23,522 | 49.6 (49-50.3) | 56.8 (56.1-57.5) |
|  | 2013 | 477,202 | 26,527 | 55.6 (54.9-56.2) | 64.3 (63.6-65) |
|  | 2014 | 481,152 | 30,618 | 63.6 (62.9-64.3) | 73.9 (73.1-74.7) |
|  | 2015 | 482,067 | 32,403 | 67.2 (66.5-67.9) | 78.2 (77.4-78.9) |
|  | 2016 | 481,359 | 33,259 | 69.1 (68.4-69.8) | 81 (80.2-81.8) |

Data represent numbers and corresponding 95% confidence intervals.
