# Supplementary material for: Sex-related differences in vitamin D testing in the Veneto Region, Italy: a retrospective analysis from 2005 to 2016
Source: Arch Osteoporos. 2024 Oct 30;19(1):105. doi: 10.1007/s11657-024-01460-w (PMC11525240; doi:10.1007/s11657-024-01460-w)
Supplement: Supplementary file 3 — Supplementary file3 (DOCX 16 KB) [file 11657_2024_1460_MOESM3_ESM.docx]

**Table 2S**. Crude and adjusted incidence rates (×1000) of blood tests performed on male and female residents for the years 2005-2016.

|  | **Year** | **Number of people who submitted**  **a blood test** | **Crude rates (CI 95%)** | **Adjusted rates (CI 95%)** |
| --- | --- | --- | --- | --- |
|  |  |  |  |  |
| *Male* | 2005 | 648 | 1.5 (1.4-1.6) | 1.4 (1.3-1.6) |
|  | 2006 | 921 | 1.8 (1.6-1.9) | 1.8 (1.6-1.9) |
|  | 2007 | 1,085 | 1.8 (1.7-2) | 1.9 (1.8-2) |
|  | 2008 | 1,592 | 2.8 (2.6-2.9) | 3 (2.8-3.1) |
|  | 2009 | 2,281 | 3.4 (3.3-3.6) | 3.8 (3.7-4) |
|  | 2010 | 3,468 | 5.1 (4.9-5.3) | 5.9 (5.6-6.1) |
|  | 2011 | 4,751 | 6.6 (6.3-6.8) | 7.7 (7.4-7.9) |
|  | 2012 | 5,829 | 7.4 (7.1-7.7) | 8.6 (8.3-8.8) |
|  | 2013 | 6,968 | 8.2 (8-8.5) | 9.7 (9.4-10) |
|  | 2014 | 8,859 | 10.3 (10-10.6) | 12.1 (11.8-12.4) |
|  | 2015 | 10,197 | 10.9 (10.6-11.2) | 12.8 (12.4-13.1) |
|  | 2016 | 10,676 | 10.8 (10.5-11.1) | 12.8 (12.4-13.1) |
|  |  |  |  |  |
| *Female* | 2005 | 3,173 | 7 (6.8-7.3) | 6.8 (6.6-7.1) |
|  | 2006 | 4,517 | 7.8 (7.6-8.1) | 7.8 (7.6-8.1) |
|  | 2007 | 5,192 | 7.9 (7.6-8.1) | 8.1 (7.8-8.4) |
|  | 2008 | 6,329 | 9 (8.7-9.3) | 9.5 (9.2-9.8) |
|  | 2009 | 9,675 | 13.2 (12.9-13.6) | 14.5 (14.1-14.8) |
|  | 2010 | 12,874 | 15.5 (15.2-15.9) | 17.2 (16.8-17.6) |
|  | 2011 | 16,333 | 17.2 (16.8-17.6) | 19.3 (18.9-19.7) |
|  | 2012 | 18,816 | 17.7 (17.3-18.1) | 19.9 (19.5-20.3) |
|  | 2013 | 21,635 | 18.3 (17.9-18.7) | 20.6 (20.2-21) |
|  | 2014 | 25,393 | 20.8 (20.4-21.2) | 23.8 (23.4-24.3) |
|  | 2015 | 27,313 | 20.1 (19.7-20.5) | 22.5 (22.1-22.9) |
|  | 2016 | 28,224 | 19.4 (19-19.8) | 21.8 (21.4-22.2) |

Data represented as number of individuals and corresponding 95% confidence intervals. Incidence was calculated as the number of new blood tests from 1,000 patients per year.
