# Supplementary material for: Sex-related differences in vitamin D testing in the Veneto Region, Italy: a retrospective analysis from 2005 to 2016
Source: Arch Osteoporos. 2024 Oct 30;19(1):105. doi: 10.1007/s11657-024-01460-w (PMC11525240; doi:10.1007/s11657-024-01460-w)
Supplement: Supplementary file 4 — Supplementary file4 (DOCX 19 KB) [file 11657_2024_1460_MOESM4_ESM.docx]

**Table 3S**. Crude and adjusted blood test prevalence rates (×1,000) on male and female residents for the years 2005-2016 by vitamin D classes (I,II,III).

|  | Crude rates class I (CI 95%) | Adjusted rates class I (CI 95%) | Crude rates class II (CI 95%) | Adjusted rates class II (CI 95%) | Crude rates class III (CI 95%) | Adjusted rates class III (CI 95%) | Crude rates class IV (CI 95%) | Adjusted rates class IV (CI 95%) |
| --- | --- | --- | --- | --- | --- | --- | --- | --- |
| Males | | | | | | | | |
| 2005 | 1.1 (1-1.2) | 1 (0.9-1.1) | 0.3 (0.3-0.4) | 0.3 (0.3-0.4) | 0.3 (0.3-0.4) | 0.3 (0.3-0.4) | 0 (0-0) | 0 (0-0) |
| 2006 | 1.8 (1.7-1.9) | 1.8 (1.7-1.9) | 0.4 (0.4-0.5) | 0.4 (0.4-0.5) | 0.3 (0.2-0.3) | 0.3 (0.2-0.3) | 0 (0-0) | 0 (0-0) |
| 2007 | 1.9 (1.8-2) | 2 (1.8-2.1) | 0.8 (0.7-0.8) | 0.8 (0.7-0.9) | 0.6 (0.5-0.7) | 0.6 (0.5-0.7) | 0 (0-0.1) | 0 (0-0.1) |
| 2008 | 2.3 (2.1-2.4) | 2.4 (2.3-2.6) | 1.3 (1.2-1.4) | 1.4 (1.3-1.5) | 1.2 (1.1-1.4) | 1.3 (1.2-1.4) | 0 (0-0) | 0 (0-0) |
| 2009 | 3 (2.8-3.2) | 3.4 (3.3-3.6) | 1.9 (1.7-2) | 2 (1.9-2.2) | 2.2 (2.1-2.4) | 2.4 (2.3-2.6) | 0 (0-0) | 0 (0-0) |
| 2010 | 4.6 (4.4-4.8) | 5.5 (5.3-5.7) | 2.9 (2.7-3.1) | 3.3 (3.1-3.4) | 3 (2.8-3.2) | 3.3 (3.1-3.5) | 0 (0-0.1) | 0 (0-0.1) |
| 2011 | 5.9 (5.7-6.1) | 7.3 (7-7.5) | 3.9 (3.7-4.1) | 4.4 (4.2-4.6) | 4.3 (4.1-4.5) | 4.8 (4.6-5) | 0.1 (0-0.1) | 0.1 (0-0.1) |
| 2012 | 7.7 (7.4-7.9) | 9.5 (9.2-9.8) | 5.6 (5.4-5.8) | 6.3 (6.1-6.5) | 4.4 (4.2-4.6) | 4.9 (4.7-5.1) | 0 (0-0) | 0 (0-0) |
| 2013 | 8.6 (8.3-8.9) | 10.8 (10.5-11.1) | 6.5 (6.3-6.8) | 7.4 (7.1-7.6) | 5.4 (5.2-5.6) | 6.1 (5.9-6.3) | 0 (0-0) | 0 (0-0) |
| 2014 | 10.9 (10.6-11.2) | 13.7 (13.4-14) | 7.7 (7.5-8) | 8.9 (8.6-9.1) | 6.3 (6.1-6.6) | 7.4 (7.1-7.6) | 0 (0-0) | 0 (0-0) |
| 2015 | 11.8 (11.5-12.1) | 14.8 (14.5-15.2) | 8.8 (8.5-9.1) | 10.2 (9.9-10.5) | 7.5 (7.2-7.7) | 8.8 (8.5-9.1) | 0 (0-0) | 0 (0-0) |
| 2016 | 9.9 (9.6-10.2) | 12.8 (12.5-13.1) | 8.7 (8.4-9) | 10.1 (9.8-10.4) | 10.5 (10.2-10.8) | 12.6 (12.3-13) | 0 (0-0.1) | 0 (0-0.1) |
|  |  |  |  |  |  |  |  |  |
| Females | | | | | | | | |
| 2005 | 5 (4.8-5.2) | 4.8 (4.6-5) | 1.7 (1.6-1.8) | 1.7 (1.5-1.8) | 1.4 (1.3-1.6) | 1.4 (1.3-1.5) | 0 (0-0) | 0 (0-0) |
| 2006 | 7.7 (7.5-8) | 7.7 (7.5-8) | 2.2 (2.1-2.4) | 2.2 (2.1-2.4) | 1.5 (1.4-1.6) | 1.5 (1.4-1.7) | 0 (0-0) | 0 (0-0) |
| 2007 | 7.6 (7.4-7.9) | 7.9 (7.6-8.1) | 3.3 (3.1-3.5) | 3.4 (3.2-3.5) | 2.4 (2.3-2.6) | 2.5 (2.3-2.6) | 0 (0-0) | 0 (0-0) |
| 2008 | 7.1 (6.9-7.3) | 7.6 (7.3-7.8) | 4.8 (4.6-5) | 5.1 (4.9-5.3) | 4.7 (4.5-4.9) | 4.9 (4.7-5.1) | 0 (0-0.1) | 0.1 (0-0.1) |
| 2009 | 9.9 (9.6-10.1) | 11.1 (10.8-11.4) | 7 (6.8-7.2) | 7.5 (7.3-7.8) | 9 (8.7-9.3) | 9.7 (9.4-10) | 0.1 (0-0.1) | 0.1 (0-0.1) |
| 2010 | 12.9 (12.6-13.2) | 14.8 (14.5-15.2) | 9.7 (9.4-9.9) | 10.5 (10.3-10.8) | 12 (11.7-12.3) | 13.2 (12.9-13.5) | 0.1 (0.1-0.2) | 0.2 (0.1-0.2) |
| 2011 | 14.8 (14.4-15.1) | 17.4 (17-17.7) | 11.9 (11.6-12.2) | 13.2 (12.9-13.5) | 16.1 (15.8-16.5) | 18 (17.6-18.3) | 0.2 (0.1-0.2) | 0.2 (0.2-0.2) |
| 2012 | 17.9 (17.5-18.2) | 21.3 (20.8-21.7) | 16.3 (16-16.7) | 18.2 (17.8-18.6) | 15.4 (15.1-15.8) | 17.3 (16.9-17.7) | 0 (0-0.1) | 0.1 (0-0.1) |
| 2013 | 18 (17.6-18.4) | 21.8 (21.4-22.2) | 17.6 (17.3-18) | 19.8 (19.4-20.2) | 19.9 (19.5-20.3) | 22.6 (22.2-23) | 0.1 (0-0.1) | 0.1 (0-0.1) |
| 2014 | 20.7 (20.3-21.1) | 25 (24.5-25.4) | 20.1 (19.7-20.5) | 22.7 (22.3-23.1) | 22.8 (22.3-23.2) | 26.2 (25.7-26.6) | 0.1 (0-0.1) | 0 (0-0.1) |
| 2015 | 21.2 (20.7-21.6) | 25.4 (24.9-25.9) | 21.1 (20.7-21.5) | 23.9 (23.4-24.3) | 24.9 (24.5-25.4) | 28.8 (28.3-29.3) | 0.1 (0-0.1) | 0.1 (0.1-0.1) |
| 2016 | 17.3 (16.9-17.6) | 21.4 (21-21.8) | 19.7 (19.3-20.1) | 22.3 (21.9-22.8) | 31.9 (31.4-32.4) | 37.1 (36.5-37.5) | 0.2 (0.1-0.2) | 0.2 (0.1-0.2) |

Data presented as blood test rates and 95% confidence intervals. Class I (<50 nmol/L), II (50-74.9 nmol/L) and III (75-149 nmol/L).
