# Supplementary material for: Sex-related differences in vitamin D testing in the Veneto Region, Italy: a retrospective analysis from 2005 to 2016
Source: Arch Osteoporos. 2024 Oct 30;19(1):105. doi: 10.1007/s11657-024-01460-w (PMC11525240; doi:10.1007/s11657-024-01460-w)
Supplement: Supplementary file 5 — Supplementary file5 (DOCX 23 KB) [file 11657_2024_1460_MOESM5_ESM.docx]

**Table 4S**. Crude and adjusted incidence rates of (×10,000) people who submitted a blood test stratified by vitamin D classes for years 2005-2016.

|  | Crude rates class I (CI 95%) | Adjusted rates class I (CI 95%) | Crude rates class II (CI 95%) | Adjusted rates class II (CI 95%) | Crude rates class III (CI 95%) | Adjusted rates class III (CI 95%) | Crude rates class IV (CI 95%) | Adjusted rates class IV (CI 95%) |
| --- | --- | --- | --- | --- | --- | --- | --- | --- |
| Males | | | | | | | | |
| 2005 | 9.7 (8.7-10.6) | 9.2 (8.3-10.1) | 2.9 (2.4-3.4) | 2.8 (2.3-3.3) | 2.5 (2.1-3) | 2.5 (2-2.9) | 0 (0-0) | 0 (0-0) |
| 2006 | 13 (11.9-14) | 13 (11.9-14) | 2.9 (2.4-3.4) | 2.9 (2.4-3.4) | 1.7 (1.3-2.1) | 1.7 (1.3-2.1) | 0.1 (0-0.1) | 0.1 (0-0.1) |
| 2007 | 12.6 (11.6-13.7) | 13.2 (12.1-14.3) | 3.6 (3.1-4.2) | 3.7 (3.2-4.3) | 2.1 (1.6-2.5) | 2.1 (1.7-2.5) | 0.1 (0-0.1) | 0.1 (0-0.2) |
| 2008 | 15.7 (14.6-16.9) | 17.2 (15.9-18.4) | 6.6 (5.9-7.4) | 6.9 (6.2-7.7) | 5.2 (4.5-5.9) | 5.3 (4.6-6) | 0.1 (0-0.2) | 0.1 (0-0.2) |
| 2009 | 18.4 (17.2-19.7) | 21.6 (20.2-22.9) | 8.6 (7.8-9.5) | 9.3 (8.4-10.2) | 7.1 (6.4-7.9) | 7.6 (6.8-8.4) | 0.1 (0-0.2) | 0 (0-0.1) |
| 2010 | 29.8 (28.2-31.4) | 36 (34.2-37.8) | 11.8 (10.8-12.8) | 13.1 (12-14.2) | 8.9 (8-9.7) | 9.5 (8.6-10.4) | 0.1 (0-0.2) | 0.2 (0-0.3) |
| 2011 | 36.2 (34.5-37.9) | 44.8 (42.8-46.8) | 17.5 (16.3-18.7) | 19 (17.8-20.3) | 11.8 (10.8-12.8) | 12.6 (11.6-13.7) | 0.1 (0-0.1) | 0.1 (0-0.1) |
| 2012 | 44.6 (42.7-46.6) | 55.2 (53-57.4) | 20.1 (18.8-21.4) | 21.3 (19.9-22.7) | 9.2 (8.3-10.1) | 9.3 (8.4-10.2) | 0 (0-0.1) | 0 (0-0) |
| 2013 | 49 (47-51.1) | 62.3 (59.9-64.6) | 22.5 (21.1-23.9) | 23.7 (22.3-25.2) | 10.8 (9.9-11.8) | 11 (10-12) | 0 (0-0) | 0 (0-0) |
| 2014 | 63.2 (60.9-65.5) | 78.6 (75.9-81.2) | 27.6 (26.1-29.1) | 29.4 (27.7-31) | 11.9 (10.9-12.9) | 12.8 (11.7-13.9) | 0 (0-0.1) | 0.1 (0-0.2) |
| 2015 | 65.5 (63.2-67.9) | 82.2 (79.5-84.9) | 30.1 (28.5-31.6) | 32.5 (30.8-34.2) | 13.1 (12.1-14.2) | 13.1 (12-14.2) | 0 (0-0) | 0 (0-0) |
| 2016 | 57.1 (54.9-59.3) | 73 (70.4-75.5) | 31.1 (29.5-32.7) | 34.4 (32.6-36.1) | 19.8 (18.6-21.1) | 20.3 (19-21.7) | 0.1 (0-0.1) | 0.1 (0-0.2) |
|  |  |  |  |  |  |  |  |  |
|  |  |  |  | Females |  |  |  |  |
| 2005 | 43.6 (41.7-45.5) | 42.3 (40.4-44.2) | 14.7 (13.6-15.8) | 14.4 (13.3-15.5) | 11.8 (10.8-12.8) | 11.5 (10.5-12.5) | 0.1 (0-0.2) | 0.1 (0-0.2) |
| 2006 | 57.5 (55.3-59.7) | 57.5 (55.3-59.7) | 13.3 (12.3-14.4) | 13.3 (12.3-14.4) | 7.4 (6.6-8.2) | 7.4 (6.6-8.2) | 0.2 (0-0.3) | 0.2 (0-0.3) |
| 2007 | 52.4 (50.3-54.4) | 53.9 (51.7-56) | 16.6 (15.4-17.7) | 16.9 (15.7-18.1) | 9.9 (9-10.8) | 10.1 (9.1-11) | 0.1 (0-0.2) | 0.1 (0-0.2) |
| 2008 | 48.3 (46.3-50.3) | 51.7 (49.6-53.8) | 23.7 (22.3-25.1) | 24.7 (23.2-26.1) | 17.9 (16.7-19.1) | 18.6 (17.4-19.9) | 0.2 (0.1-0.3) | 0.2 (0.1-0.3) |
| 2009 | 67.8 (65.5-70.2) | 76.1 (73.6-78.6) | 33.4 (31.8-35.1) | 35.7 (34-37.4) | 31 (29.5-32.6) | 33 (31.3-34.6) | 0.1 (0-0.3) | 0.2 (0-0.3) |
| 2010 | 83.1 (80.5-85.7) | 95.1 (92.3-97.9) | 40.1 (38.3-41.9) | 42.8 (41-44.7) | 31.6 (30-33.2) | 34 (32.3-35.7) | 0.3 (0.1-0.4) | 0.3 (0.2-0.5) |
| 2011 | 89.6 (87-92.3) | 104.1 (101.1-107) | 43.9 (42-45.8) | 47.1 (45.1-49.1) | 38.2 (36.4-39.9) | 41.1 (39.2-42.9) | 0.3 (0.2-0.5) | 0.4 (0.2-0.6) |
| 2012 | 101 (98.2-103.8) | 117.8 (114.7-121) | 49.2 (47.2-51.2) | 52.3 (50.2-54.4) | 26.6 (25.1-28) | 28.8 (27.3-30.4) | 0.1 (0-0.1) | 0.1 (0-0.1) |
| 2013 | 99.5 (96.6-102.3) | 117 (113.8-120.1) | 52.1 (50.1-54.2) | 54.8 (52.7-57) | 31.4 (29.8-33) | 34 (32.3-35.7) | 0 (0-0.1) | 0.1 (0-0.1) |
| 2014 | 115.3 (112.3-118.3) | 135.9 (132.6-139.3) | 58.9 (56.7-61.1) | 64 (61.7-66.4) | 33.7 (32-35.3) | 38.1 (36.3-39.9) | 0.1 (0-0.3) | 0 (0-0.1) |
| 2015 | 110.4 (107.4-113.3) | 127.9 (124.6-131.1) | 56.6 (54.5-58.7) | 60.1 (57.9-62.4) | 33.9 (32.2-35.5) | 36.9 (35.2-38.7) | 0.1 (0-0.2) | 0.1 (0-0.2) |
| 2016 | 92.4 (89.7-95.1) | 109.8 (106.7-112.8) | 56.7 (54.6-58.8) | 59.3 (57.1-61.5) | 44.6 (42.8-46.5) | 48.5 (46.5-50.6) | 0.2 (0.1-0.3) | 0.1 (0-0.3) |

Data presented as incidence rates and 95% confidence intervals (CI). Class I (<50 nmol/L), II (50-74.9 nmol/L) and III (75-149 nmol/L).
